# Microbial N_2_O consumption in and above marine N_2_O production hotspots

**DOI:** 10.1038/s41396-020-00861-2

**Published:** 2020-12-21

**Authors:** Xin Sun, Amal Jayakumar, John C. Tracey, Elizabeth Wallace, Colette L. Kelly, Karen L. Casciotti, Bess B. Ward

**Affiliations:** 1 Department of Geosciences, Princeton University, Princeton, NJ 08544 USA; 2 Department of Earth System Science, Stanford University, Stanford, CA 94305 USA

**Keywords:** Water microbiology, Biogeochemistry, Microbial ecology

## Abstract

The ocean is a net source of N_2_O, a potent greenhouse gas and ozone-depleting agent. However, the removal of N_2_O via microbial N_2_O consumption is poorly constrained and rate measurements have been restricted to anoxic waters. Here we expand N_2_O consumption measurements from anoxic zones to the sharp oxygen gradient above them, and experimentally determine kinetic parameters in both oxic and anoxic seawater for the first time. We find that the substrate affinity, O_2_ tolerance, and community composition of N_2_O-consuming microbes in oxic waters differ from those in the underlying anoxic layers. Kinetic parameters determined here are used to model in situ N_2_O production and consumption rates. Estimated in situ rates differ from measured rates, confirming the necessity to consider kinetics when predicting N_2_O cycling. Microbes from the oxic layer consume N_2_O under anoxic conditions at a much faster rate than microbes from anoxic zones. These experimental results are in keeping with model results which indicate that N_2_O consumption likely takes place above the oxygen deficient zone (ODZ). Thus, the dynamic layer with steep O_2_ and N_2_O gradients right above the ODZ is a previously ignored potential gatekeeper of N_2_O and should be accounted for in the marine N_2_O budget.

## Introduction

Nitrous oxide (N_2_O) is not only a greenhouse gas with about 300 times greater radiative forcing per mole than carbon dioxide, it is also the dominant ozone-depleting agent emitted in the 21st century [1]. The N_2_O concentration in the atmosphere is increasing [2], and the rate of N_2_O emission is accelerating [3]. From 2007 to 2016, the ocean contributed 20% of global N_2_O emissions, and 35% of the natural sources on average [4]. N_2_O cycling in the ocean thus has the potential to exacerbate climate change, as well as being affected by associated chemical changes, such as ocean acidification [5]. The most intense sources and sinks of N_2_O in the ocean occur in oxygen minimum zones (OMZs) [6], which are marine regions characterized by a sharp O_2_ gradient (oxycline) overlying an oxygen deficient zone (ODZ) where O_2_ concentration is below the detection limit of a switchable trace oxygen (STOX) sensor (10 nM) [7]. There are multiple biological sources of N_2_O [8–10], but there is only one major biological sink [11] (Fig. S1): the reduction of N_2_O to N_2_ by N_2_O-consuming microbes using the nitrous oxide reductase enzyme (N_2_OR). The possibility of N_2_O fixation has been suggested [12], but its mechanism is yet to be determined. N_2_O consumption in oxic waters, including the oxic layer of OMZs, has been ignored because this process was assumed to be part of the complete denitrification pathway (reduction of nitrate to N_2_ gas) and to be restricted to suboxic/anoxic environments (such as ODZs) [13]. However, the oxic surface layer and the oxycline of OMZs above the ODZ could be of vital importance in regulating N_2_O emissions if N_2_O consumption occurs there. N_2_O concentration in the oxycline or the anoxic ODZ of OMZs can be ≥10-fold higher than atmospheric saturation at the air–sea interface [11]. If not consumed in situ, this excess N_2_O could diffuse through the oxycline and the surface layer or be upwelled into the surface where it can exchange with the atmosphere.

The functional marker of the operon encoding N_2_OR, *nosZ*, has been used as a proxy for the presence of N_2_O-consuming microbes. Recent detection of *nosZ* genes and transcripts in oxic seawater [14, 15] implies the potential for N_2_O consumption there. The presence of genes and transcripts, however, does not guarantee the successful translation or activity of the enzyme. Direct rate measurements are required to determine whether this microbial potential actually results in N_2_O consumption. Here, the abundance and community composition of N_2_O-consuming microbes were determined by qPCR and microarray, respectively. N_2_O-consuming microbes that contain only *nosZ* (i.e., none of the other genes in the complete denitrification pathway) are of particular interest, because their activity results in net N_2_O consumption. Based on the analysis of 652 draft or complete microbial genomes with one or more dentification genes, *nosZ*-only microbes are overrepresented among these isolates from the ocean compared to other ecosystems [16]. N_2_O consumption rates were also measured under a matrix of controlled N_2_O and O_2_ concentrations in ~3000 samples collected from oxic and anoxic depths in the Eastern Tropical North Pacific (ETNP) OMZ, one of the three major oceanic OMZs. O_2_ tolerance and substrate kinetics of N_2_O consumption were determined and used to estimate in situ N_2_O consumption and production rates, which reflect the in situ conditions more accurately than directly using measured rates from incubation experiments without correcting for substrate additions.

## Results and discussion

### N_2_O consumption rates and N_2_O-consuming microbes in the OMZ

**Fig. 1:**
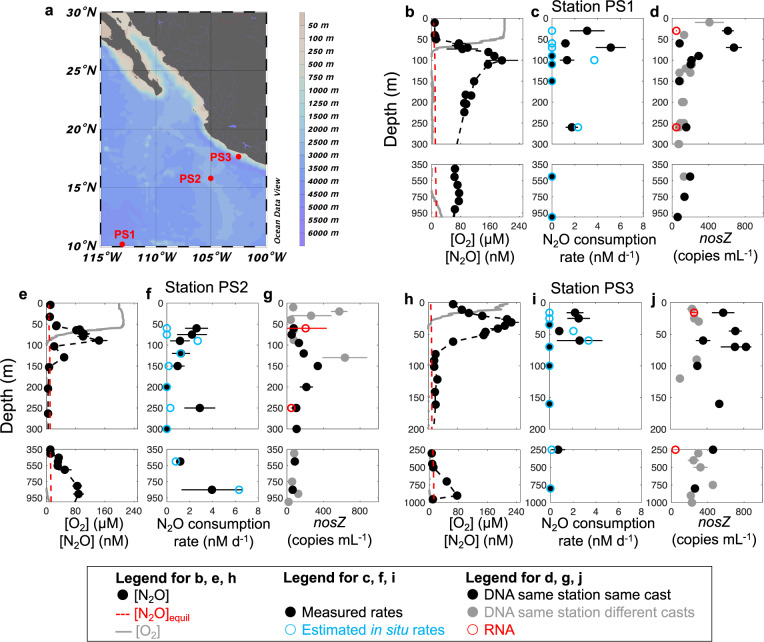
Locations of sampling stations and depth profiles of O_2_, N_2_O, N_2_O consumption rates, and *nosZ* copy numbers at stations PS1, PS2, and PS3 of the ETNP OMZ. **a** Scale bar indicates depth of water. **b**, **e**, **h** Note breaks in the *y*-axes. Gray lines indicate O_2_ concentration, black dots indicate N_2_O concentration from (Kelly et al. accepted) [37] and red dashed lines indicate the N_2_O concentration at equilibrium with the atmosphere [38]. **c**, **f**, **i** Black circles with black bars indicate measured N_2_O consumption rates with standard errors (also shown in Table S2). Error bars are standard errors of linear regression slope calculated from 15 point time-course incubations (three replicates at 5 time points). Blue circles indicate estimated in situ rates, which will be discussed in the “Estimated in situ N_2_O consumption and production rates” section. **d**, **g**, **j** Filled black circles indicate copy number of *nosZ* DNA in samples collected from the same casts from which rates were measured, filled gray circles indicate *nosZ* DNA from other casts at the same stations sampled within the same week, and red circles indicate copy numbers of *nosZ* RNA from the same casts. None of the RNA error bars overlapped with the zero line. Error bars are standard deviation (*n* = 3). Error bars are not shown if smaller than the symbol.

Potential N_2_O consumption rates (hereafter “measured rates”) were determined in March and April 2018 at three stations in the ETNP OMZ (Fig. 1a). Anoxic incubations were amended with standard additions of (^15^N)_2_O tracer with a final concentration of 50 nM at stations PS1 (on the west margin of the OMZ), PS2 (the open ocean station) and PS3 (the coastal station). Measured N_2_O consumption rates varied from zero to 5.1 nM d^−1^ at different depths (Fig. 1c, f, i). Measured rates in oxygen deficient waters were on the same order of magnitude (a few nM d^−1^) as previously measured rates in the ETNP, but lower than rates at one coastal station in that study [6], indicating high variability of N_2_O cycling in the coastal regions as previously suggested [17]. Notably, significant N_2_O consumption rates were measured in these anoxic incubations, even in samples collected from the oxycline and the oxygenated surface ocean (in situ [O_2_] up to 199 µM, Tables S1,  S2), where N_2_O-consuming microbes were present and the *nosZ* gene was transcribed (Fig. 1d, g, j). In the upper water column, measured N_2_O consumption rates were highest in the upper oxycline, above the peak of in situ N_2_O concentrations at each station (Fig. 1b, e, h), and the rate maximum at station PS2 was detected in samples collected from 60 m where in situ O_2_ was 173.9 µM (Tables S1,  S2). The rates of N_2_O consumption measured in surface waters stripped of oxygen were similar to or larger than rates measured in the ODZ. The larger consumption rates in the oxic layer given the same N_2_O and O_2_ concentrations as the ODZ layer might be due to more available dissolved organic matter at the shallower depths compared to the deeper ODZ layer [18] and/or the presence of different microbial communities at these depths.

**Fig. 2:**
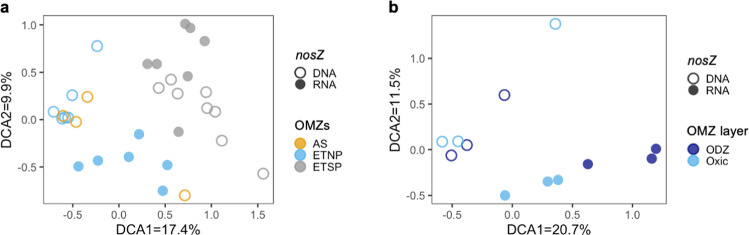
Detrended correspondence analysis (DCA) of *nosZ* DNA and *nosZ* RNA transcripts. **a** N_2_O-consuming microbes from all three major OMZs (ETNP, ETSP (Eastern Tropical South Pacific) and AS (Arabian Sea)). Arabian Sea samples include two samples from the oxic layer above the ODZ and two inside the ODZ at station 1 (19N, 66E) collected on a previous cruise [34], and ETSP samples include four samples from oxic layers and four inside ODZs. ETSP data were obtained from a previous study [15]. **b** *nosZ* data from the ETNP only.

N_2_O-consuming microbes in the oxic surface water and oxycline were at least as abundant (DNA) and transcriptionally active (in terms of *nosZ* RNA abundance) as those in ODZs (Fig. 1d, g, j). Diverse archetypes of N_2_O-consuming microbes were detected in the oxic water above the ODZ at three stations in the ETNP, one station in the Arabian Sea, and were previously detected at two stations in the Eastern Tropical South Pacific (ETSP) (Fig. 2a) using a *nosZ* microarray. The microarray is not quantitative, but it can detect diverse, low abundance microbes from environmental microbial assemblages. Even though the microarray cannot represent every *nosZ* variant, the probe set (which includes marine, salt marsh, and terrestrial representatives) allowed us to determine that the community composition of the ETSP N_2_O-consuming microbial assemblages differs from the other two OMZs (Fig. 2a). Within the ETNP, the community composition of *nosZ* microbes at the RNA level differed between oxic and ODZ waters (Fig. 2b). The detection of N_2_O consumption in samples from the oxycline and the oxygenated surface seawater of the ETNP OMZ, and the presence of N_2_O-consuming microbes in all three major OMZs (Fig. 2a), indicate that microbes in the oxic layer above ODZs have the capacity to consume N_2_O at least under anoxic incubation conditions.

### O_2_ tolerance of N_2_O-consuming microbes

**Fig. 3:**
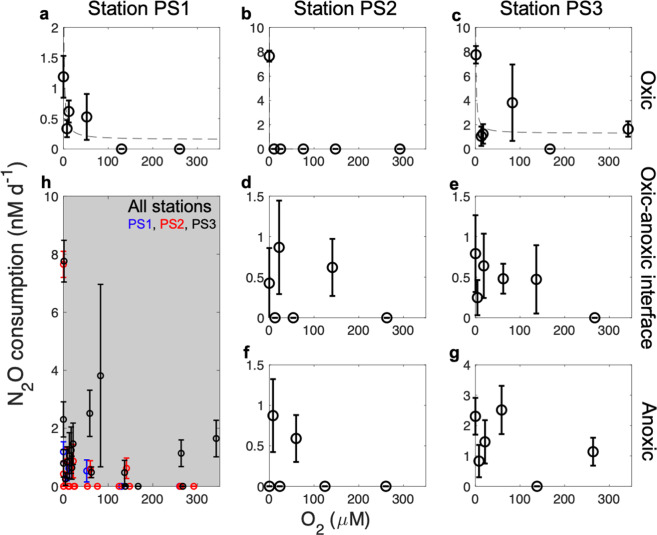
O_2_ tolerance of N_2_O consumption at three stations of the ETNP OMZ. Samples were from (**a**, **b**, **c**) the oxic layer, (**d**, **e**) the top of the ODZ, which is the oxic–anoxic interface, and (**f**, **g**) anoxic ODZ core at stations (**a**) PS1, (**b**, **d**, **f**) PS2, and (**c**, **e**, **g**) PS3. Sample depths and in situ O_2_ concentrations are shown in Table S3. O_2_ concentrations on the *x*-axis were measured in incubation bottles using PyroScience optical O_2_ sensors. Dashed lines are fitted inhibition curves (see “Methods”). No half-inhibition constant (*K*_*i*_) is significantly different from zero. The gray subplot (**h**) includes all data from the other plots (station PS1: blue, PS2: red, PS3: black). Error bars for each rate are standard errors of linear regression slope calculated from 15 point time-course incubations (three replicates at 5 time points). Error bars are not shown if smaller than the symbol or when rates are not significantly different from zero.

The O_2_ tolerance of N_2_O-consuming microbes was determined by incubating seawater under a range of O_2_ conditions and measuring the O_2_ concentration corresponding to each rate using Pyroscience optical O_2_ sensors. N_2_O consumption rates were highest when O_2_ was lowest in almost all incubations (Fig. 3). The highest rates were measured in anoxic incubations with samples from the oxic seawater (Fig. 3b, c and Table S3), and surprisingly, these rates were much higher than rates measured in samples collected from anoxic depths at the same station (Fig. 3d–g). These results suggest that N_2_O-consuming microbes found in the oxic layer have the potential to metabolize N_2_O more rapidly than anaerobic organisms when conditions become anoxic (Fig. 1d, g, j). At stations PS1 and PS3, the higher potential of N_2_O consumption also corresponds to higher *nosZ* gene copy numbers in the oxic layer (Fig. 1d, j). N_2_O consumption rates in samples from oxic seawater, but not the ODZ, decreased sharply with increasing O_2_, indicating that N_2_O-consuming microbes from oxic seawater (Fig. 3a–c) were more sensitive to O_2_ than those from anoxic seawater (Fig. 3d–g). Although N_2_O consumption in samples from the oxic layer did not occur at high O_2_ concentrations, it started rapidly (≤1 day) after transitioning from oxic in situ conditions to anoxic incubation conditions (Fig. S2). The speed of this response might be due to the growth of N_2_O-consuming microbes, fast enzyme (N_2_OR) translation, or a response by already translated N_2_OR in the oxic seawater prior to sampling.

**Fig. 4:**
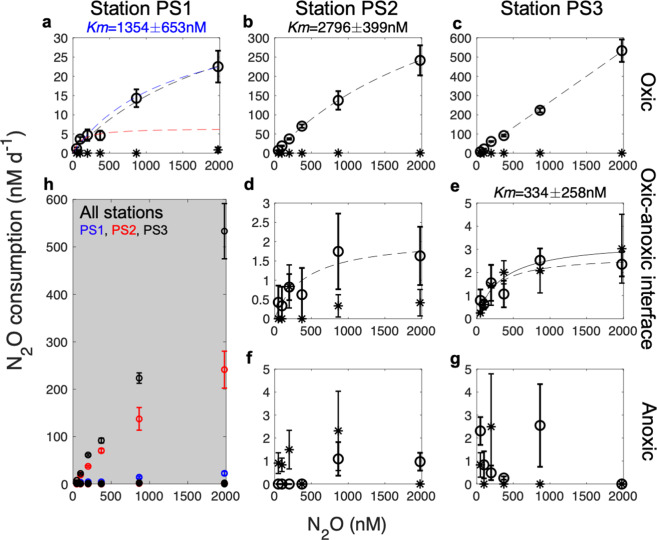
N_2_O kinetics of N_2_O consumption rate at three stations of the ETNP OMZ. Samples were from (**a**, **b**, **c**) the oxic layer, (**d**, **e**) the top of the ODZ which is the oxic–anoxic interface, and (**f**, **g**) anoxic ODZ core at stations (**a**) PS1, (**b**, **d**, **f**) PS2, and (**c**, **e**, **g**) PS3. The gray subplot (**h**) includes all data from the other plots (station PS1: blue, PS2: red, PS3: black). Open circles indicate incubations without O_2_, and dashed lines are Michaelis–Menten curves fitted to these open circles. Stars indicate incubations with O_2_ additions (final O_2_ concentrations measured in incubation bottles, in situ O_2_ and sample depths shown in Table S3), and solid lines are Michaelis–Menten curves fitted to these stars. The blue line in (**a**) is curve fitted to all except the 4th datapoint, and the red line in (**a**) is curve fitted to the first four datapoints. Although the increase in rate with increasing N_2_O concentration was significant in six sets of kinetic experiments, only three curves resulted in significant *K*_*m*_ values (*K*_*m*_ corresponding to the blue dashed line in **a**, the dashed line in **b**, and the solid line in **e**). Error bars for each rate are standard errors of linear regression slope calculated from 15 point time-course incubations (three replicates at 5 time points). Error bars are not shown if smaller than the symbol.

While our rate measurements suggest that N_2_OR is not active under oxic conditions, molecular data obtained here (Fig. 1d, g, j) and previously [15] show that both *nosZ* RNA and N_2_OR can be synthesized under oxic conditions. This phenomenon is seen in other environments as well, for example, an obligate aerobe from soil requires O_2_ to initiate *nosZ* expression, and can use the N_2_OR enzyme to consume N_2_O to survive temporary anoxia [19]. Another microbial culture continually makes N_2_OR and stores the enzymes inside their cells under oxic conditions, which was proposed as a “bet-hedging” strategy by this facultative anaerobe to allow for a rapid transition into anoxic environments [20]. Regardless of the mechanism of the rapid response, our results indicate that microbes from oxic seawater have the genetic potential to consume N_2_O, that the consumption was not limited by organic matter supply (in situ limitation by organic matter would prevent the observed increase in rate with increasing N_2_O concentration, Fig. 4a–c) and that they could consume N_2_O under anoxic conditions. These anoxic conditions could occur at a small scale in otherwise oxic water; for example they could be associated with phytoplankton colonies [14] and other particles [21, 22], especially in the productive and dynamic oxyclines of the OMZ with strong O_2_ and N_2_O gradients at shallow depths.

### Substrate affinity of N_2_O-consuming microbes

In incubations without O_2_, measured *K*_*m*_ values (half-saturation constants of the Michaelis–Menten curve) (Fig. 4) for N_2_O consumption were in excess of in situ N_2_O concentrations at every station and depth (Fig. 1b, e, h), indicating that the in situ N_2_O concentrations were too low to saturate the N_2_O consumption rate. Notably, the potential maximum rate of N_2_O consumption in the oxic layer, upon removal of O_2_, was much higher than that in the ODZ at the same station (Fig. 4), and the substrate affinities of N_2_O-consuming microbes were distinct between oxic and anoxic depths (Fig. 4). Consistently, the community composition of N_2_O-consuming microbes at the RNA level was also different between oxic layers and anoxic ODZs of the ETNP (Fig. 2b). These results indicate that different kinetics parameters should be applied to the estimate of the anoxic N_2_O sink and the newly discovered potential N_2_O sink in the oxic layer. The difference in substrate affinities is likely due to diverse N_2_O-consuming microbes occupying different niches, because their affinities for N_2_O can vary by two orders of magnitude as shown in pure cultures [23]. This difference might be obscured when microbes in different layers are mixed up by physical processes such as upwelling and eddies.

Significant *K*_*m*_ for N_2_O consumption could be determined for the oxic layer at station PS2 and the oxic–anoxic interface at station PS3 (Fig. 4b, e). The lack of Michaelis–Menten kinetics in samples from anoxic ODZs (Fig. 4f, g) implies that factors (e.g., organic matter) other than the added substrate were limiting N_2_O consumption. *K*_*m*_ for the oxic layer at station PS3 was likely larger than that of station PS2, because the rate was not saturated even at the maximum N_2_O concentration (Fig. 4c). As for station PS1, the decreasing rate at the 4th datapoint suggests a mixture of N_2_O-consuming microbes with different substrate affinities (Fig. 4a). Although not significant, *K*_*m*_ was 110 (±230) nM when only including the first four datapoints. When excluding the 4th datapoint, *K*_*m*_ was 1354 (±653) nM for the oxic layer at station PS1. Larger *K*_*m*_ values in samples from the oxic layer indicate that microbes there have lower affinity for N_2_O.

Notably, *nosZ* archetypes closely related to *A. dehalogenans* were among the top five most abundant archetypes at almost all examined depths from the ETNP (Tables S4,  S5), ETSP [15], and Arabian Sea (Table S6), implying the importance of *A. dehalogenans*-like N_2_O-consuming microbes in both oxic layers and ODZs of all major OMZs. Furthermore, the *K*_*m*_ (1.3 μM) of *Anaeromyxobacter dehalogenans* determined in cultures [23] was within the range of the *K*_*m*_ values determined in this study (2.8 μM in the oxic layer and 0.3 μM at the top of the ODZ, Fig. 4). Different overall community composition, but similar affiliation of the most abundant archetypes, implies that the difference in community composition between the oxic and ODZ assemblages in the ETNP (Fig. 2b) and the difference between the ETSP and the other two OMZs (Fig. 2a) results from a diversity of low abundance microbes rather than a few abundant clades. The microarray probes cannot identify exact species, but can differentiate among archetypes representing unknown microbes, such as the *A. dehalogenans*-like N_2_O-consuming types that were detected in most of these samples. The vital role of *A. dehalogenans*-like microbes and other microbes in the same clade has been demonstrated in soils [24, 25]. Because *A. dehalogenans* possesses *nosZ* but no other denitrification genes [26], *A. dehalogenans*-like microbes may decouple N_2_O consumption from its production, resulting in a net N_2_O sink at depths where they dominate N_2_O-consuming assemblages.

In addition to anoxic incubations, we also examined N_2_O consumption kinetics in oxic incubations. Consistent with the low O_2_ tolerance of N_2_O-consuming microbes, especially in the oxic layers (Fig. 3), the kinetics of N_2_O consumption could not be determined under most incubations with O_2_ additions because N_2_O consumption rates were not detected (Fig. 4). Only samples from the oxic–anoxic interface at station PS3 showed Michaelis–Menten kinetics in the oxic incubation (Fig. 4e), likely because the O_2_ addition in this incubation (4.5 μM, Table S3) was less than all the other oxic incubations (8.1–342.0 μM, Table S3) and N_2_O-consuming microbes in ODZs had higher tolerance to increasing O_2_ concentration than those from the oxic layer (Fig. 3e). The O_2_ concentration (≥4.5 μM) allowing the occurrence of N_2_O consumption (N_2_O→N_2_) here was much higher than the previously determined threshold (0.2–0.3 µM) for denitrification (NO_2_^−^→ N_2_O or N_2_) [27], which might be due to differential O_2_ sensitivities of microbes possessing different parts of the denitrification pathway. Variable oxygen sensitivities have also been observed in O_2_ thresholds for N_2_O production from NO_2_^−^ and NO_3_^−^, the latter showing a higher tolerance to O_2_ [10, 28]. In addition, unlike the presence of *nosZ* in the oxic layers, the group of denitrifiers represented by nitrite reductase genes (*nirK* and *nirS*) were very rare in the oxic layers [29]. These observations suggest that denitrification can be carried out in a modular fashion by independent organisms possessing different segments of the pathway [16], rather than one process of coupled reactions that occur without exchange of intermediates. Modular denitrification may have implications for the interpretation of classical isotope pairing experiments.

### Estimated in situ N_2_O consumption and production rates

Rates of biogeochemical processes inferred from incubation experiments can be biased away from in situ values due to the dependence of rates on substrate concentrations and other environmental factors (e.g., O_2_ concentration), which often differ between in situ and incubation conditions. However, the new quantitative information on N_2_O consumption kinetics and effects of environmental factors like O_2_ derived here can be used to estimate in situ rates. Using these kinetic parameters most in situ N_2_O consumption rates were inferred to be zero in oxic layers (Fig. 1c, f, i) based on the high sensitivity of those microbes to O_2_ (Fig. 3a–c). The exception was 90 m at station PS2 where the in situ O_2_ concentration (4.4 µM, Table S1) was likely to be low enough to allow N_2_O consumption based on similar kinetics in anoxic incubations and incubations at PS3 with 4.5 µM O_2_ (Fig. 4e). In situ N_2_O consumption rates in anoxic ODZs were simulated by the Michaelis–Menten equation using the *K*_*m*_ value determined here (Fig. 4e) and the in situ N_2_O concentrations (Fig. 1b, e, h). Measured N_2_O consumption rates had maxima in the upper oxycline above the N_2_O concentration peaks at all three stations, but the maxima in estimated in situ rates at these stations occurred at or below the oxic–anoxic interface, and the highest rate at station PS2 (6.3 nM d^−1^) was at 850 m, the lower edge of the ODZ (Fig. 1c, f, i). The secondary peak of N_2_O consumption inside the ODZ at station PS2 was greatly reduced after corrections were made using in situ N_2_O concentrations due to low in situ N_2_O concentrations (Fig. 1e). The peak inside the ODZ at station PS1, however, was larger after correction because the in situ N_2_O concentration (~80 nM, Fig. 1b) was higher than that in incubations (50 nM). The persistently high N_2_O concentration in the ODZ core at station PS1 reflects the slow N_2_O removal by denitrification at the margin of the OMZ. The lack of a SNM, a typical feature for anoxic ODZs, at station PS1 (Fig. S3) is also consistent with its position at the oceanic edge of the OMZ. The difference between measured rates and kinetics-corrected in situ rates indicates the need for more information on the kinetics of N_2_O consumption under different environmental conditions and in different OMZ regions.

**Fig. 5:**
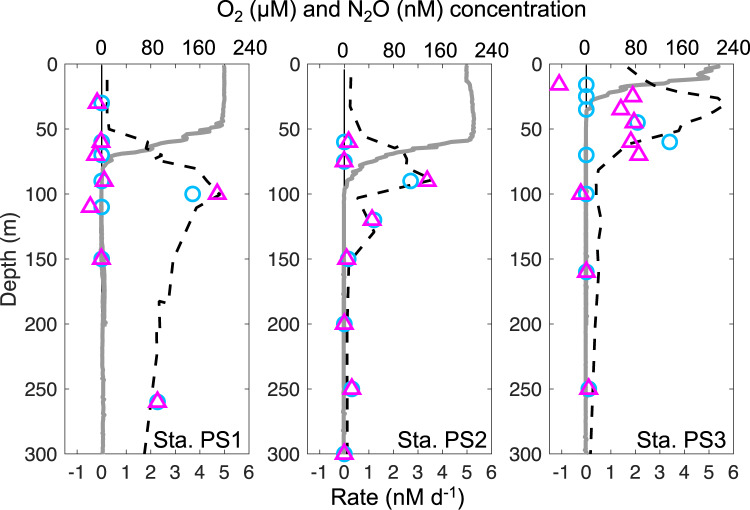
Depth profiles of modeled N_2_O production rates (magenta triangles) at stations PS1, PS2, and PS3 of the ETNP OMZ plotted together with observed O_2_ (µM, gray lines), N2O (nM, black dashed lines), and estimated in situ N_2_O consumption rates (blue circles) from Fig. 1. Production rates were calculated by subtracting the advection and diffusion of N_2_O from in situ consumption rates assuming a steady state.

In situ N_2_O production rates were modeled from estimated in situ N_2_O consumption rates, N_2_O concentrations, advection, and diffusion using a 1-D steady-state framework (Fig. 5), which reflects a weak lateral advection and upwelling scenario as in a previous study [6]. Production and consumption rates were mostly balanced but were decoupled at the sharp N_2_O concentration gradient at station PS3. The decoupling of the production and consumption was due to the strong N_2_O fluxes from physical processes (i.e., advection and diffusion) in the sharp N_2_O gradient coinciding with the sharp O_2_ gradient. This decoupling was not reported previously because measurements of N_2_O consumption were all below the bottom of the upper oxycline [6]. Notably, the modeled production rates in the oxic layer at stations PS1 and PS3 (Fig. 5) with 199.0 and 89.9 µM in situ O_2_ were negative considering advection, diffusion and zero estimated consumption rates (Table S1). Since the production rate cannot be negative, this analysis suggests that N_2_O consumption occurs at least sometimes at these depths to balance the N_2_O flux from physical processes (assuming steady state). Consistent with the model results, N_2_O consumption rates were detected when oxygen is above the 4.5 µM threshold especially at station PS3 (Fig. 3c, e, j). The significant rates under oxic conditions might imply more micro-anoxic sites (e.g., particulate organic matter) at the coastal station. Although we chose 4.5 µM as a conservative oxygen threshold here, different thresholds for N_2_O consumption need to be determined for different environmental conditions in future studies. Particles and other microsites might disintegrate during sampling and purging, so the in situ N_2_O consumption rates in the oxic layer were potentially underestimated by the incubation experiments.

### Implications for the oceanic N_2_O budget

The annual N_2_O emission rate of the ocean is 3–5 Tg-N yr^−1^ based on recent estimates [4, 30]. Developing an understanding of what controls the major biological sink of N_2_O is vitally important to better constrain estimation of the highly uncertain [17] marine N_2_O budget. Using direct rate measurements, we demonstrated the ability of microbial assemblages in the oxic layer above N_2_O production hotspots to consume N_2_O under anoxic conditions and quantified the rate dependence on N_2_O and O_2_ concentrations. The potential N_2_O consumption rate in oxic seawaters was at least two orders of magnitude faster than that in ODZs under favorable conditions (i.e., low O_2_ and high N_2_O concentrations). Even though N_2_O is unlikely to be consumed when surface seawater is saturated with O_2_, N_2_O consumption started rapidly after switching to anoxic conditions. N_2_O consumption also occurred in the oxycline where O_2_ concentration was low and N_2_O concentration was high. High N_2_O concentrations in the oxycline are attributed to production from both denitrification and nitrification, with dentification as the main source [10]. Assuming anoxia occurs transiently in the layer above the ODZ, the consumption rate (0.7–10.3 nM d^−1^) estimated from measured rates using in situ N_2_O concentrations (Table S1) are on the same order of magnitude as N_2_O production rates measured in the ETNP OMZ [10]. These rates suggest a potential gross N_2_O sink in the layer above the ODZ of all OMZs of 0.02–0.32 Tg-N yr^−1^ assuming a 10 cm transiently anoxic depth interval of this layer with a volume of 3.04 × 10^11^ m^3^ [31]. Although anoxia is unlikely to occur in such a large area at once, this potential gross sink of N_2_O is considerable relative to an annual oceanic N_2_O emission of 3–5 Tg-N yr^−1^ [4, 30].

Anoxic conditions could occur in microsites such as particles [21] and phytoplankton aggregates [14] in the oxic layer. *nosZ* transcripts were found to be highly enriched in particle-associated fractions compared to free-living fractions in the ETNP OMZ [22]. Additionally, eddies, upwelling and other dynamic mixing events could lead to the shoaling of low oxygen, high N_2_O seawater, and N_2_O-consuming microbes [32]. O_2_ concentrations in the oxycline above the ODZ of the ETNP varied from nearly 100 µM to below detection within days or weeks based on both Argo float data (Fig. S4) and conductivity–temperature–depth (CTD) Seabird data (Fig. S5). Thus, microbes in the surface layer or subsurface microbes being brought to the surface layer could consume a portion of N_2_O before it escapes into the atmosphere when surface anoxia occurs.

The detection of viable N_2_O-consuming microbes in the upper oxyclines of all three OMZs implies a potential role for unconventional *nosZ*-containing microbes in regulating the N_2_O budget. The presence of these microbes in all three OMZs and their potential N_2_O sink raises the necessity of quantifying this potential in the other two OMZs. The substrate kinetics and biological information obtained in this study provide previously lacking parameters for the characterization of N_2_O consumption in marine N_2_O models. Applying the new O_2_ threshold obtained here for N_2_O consumption (4.5 µM) to a mechanistic 1-D biogeochemistry model [6] produces N_2_O peaks and O_2_ profiles similar to our measurements at the open ocean station when the O_2_ threshold for N_2_O production via denitrification was also increased (to 20 µM [10, 28]) (Fig. S6). The O_2_ thresholds for N_2_O production and consumption along the redox gradient of the ocean need further investigation, but the high O_2_ tolerance of N_2_O production from denitrification in the modified model is consistent with previous experimental results showing the persistence of N_2_O production from NO_3_^−^ at 7 and 23 µM O_2_ in the ETNP [10] and ETSP [28], respectively. Further kinetics and molecular experiments are required to investigate the co-occurrence of microbes with different substrate kinetics in the same sample (Fig. 4a) and the spatial variation of microbial communities for both N_2_O consumption and production. The OMZs are not only the most intense N_2_O cycling regions [6, 10], but also contribute to a large seasonal variation to the global N_2_O emissions [30]. Thus, the findings here will not only improve estimates of these N_2_O sinks, but also will improve estimates of N_2_O sources, two crucial variables to constrain in a changing ocean.

## Methods

### Sampling, incubations, and rate measurements

The sampling sites are within the ETNP OMZ, one of the three major OMZs in the world. We sampled at three stations (OMZ margin station PS1, open ocean OMZ station PS2, and coastal OMZ station PS3; Fig. 1a) in March and April 2018 on board R/V Sally Ride (Cruise ID: SR 1805). The three sampling stations represent a transect from offshore to onshore, along a gradient from low to high productivity, and from the oceanic edge of the OMZ to the intense ODZ of the coastal station. Station PS1 is on the margin of the ETNP OMZ, so oxygen intrusion events likely occur below the upper oxycline at this station. Station PS1 does not have a secondary nitrite maximum (SNM), while stations PS2 and PS3 both have SNM in their ODZs (Fig. S3). PS1 and PS2 are open ocean stations, and PS3 is a coastal station. Station PS3 has a very shallow oxycline close to the surface of the ocean. This shallow oxyline is probably due to the intense heterotrophic respiration fueled by the primary production (implied by chlorophyll concentrations of 3 µg/L at 10 m) at the surface.

Twelve 30 L Niskin bottles on a rosette with a CTD profiler were used to collect seawater from different depths while recording temperature, pressure, salinity, chlorophyll, and in situ O_2_ concentration with both Seabird (Sea-Bird SBE 9, Sea-Bird Electronics, Bellevue, NA) and STOX sensors [7]. Samples for measurements of in situ N_2_O concentration were collected from Niskin bottles into 160 mL bottles after overflowing three times and preserved with saturated HgCl_2_. N_2_O concentrations were measured on an isotope ratio mass spectrometer based on the major ion (*m*/*z* = 44) peak area [33]. Particulate material for microbial DNA and RNA analysis was collected by filtering up to 4 L seawater from Niskin bottles through Sterivex filters (0.22 µm). Filters were flash frozen in liquid N_2_ on board, and then preserved at −80 °C until DNA and RNA extraction in the lab. Seawater samples for determining N_2_O consumption rate were collected into 320 mL ground glass-stoppered glass bottles after overflowing three times to minimize O_2_ contamination. Seawater (8 mL) was then aliquoted into 12 mL exetainers inside a N_2_ flushed glove bag to leave a 4 mL headspace for purging. After sealing in the glove bag, exetainers were purged with helium for 5 min to reach anoxia for depth profiles shown in Fig. 1 and kinetics determined under anoxic conditions shown in Fig. 4. (^15^N)_2_O tracer (Cambridge Isotope Laboratories, purity ≥98%) was added as gas into each exetainer using a helium-flushed gas-tight glass syringe to reach a final concentration of 50 nM N_2_O (standard additions shown in Figs. 1,  3) or varying from 50 to 1978 nM N_2_O for kinetics experiments in Fig. 4. A 50 nM N_2_O addition was made to ensure the produced N_2_ was detectable. A set of 15 exetainers incubated in a time series (triplicates for each time point, 5 time points in total including three time zero bottles as abiotic controls) was used to determine a single rate. Incubations were sampled approximately every 12 h for 2 days and were terminated by adding 0.05 mL of 50% (w/v) ZnCl_2_ following previous procedures [6]. The amount and isotopic composition of N_2_ in each exetainer was measured on a mass spectrometer (Europa Scientific 20-20, Crewe, UK), and the rate of N_2_O consumption was calculated from the linear regression of the excess of ^30^N_2_ over the incubation time following the previous method [34].

N_2_O consumption rates were measured at 10 depths at each station, including oxic surface water, upper and lower oxycline, top of the anoxic ODZ (oxic–anoxic interface), and core of the ODZ. The position of the upper oxycline varies among the three stations (station PS2: 59–96 m; station PS3: 48–84 m; station PS3: 0–35 m). The lower oxycline starts at around 700 m for station PS1, 850 m for station PS2, and 860 m for station PS3. N_2_O kinetics and O_2_ tolerance experiments were performed in the oxic layer, at the top of the ODZ (oxic–anoxic interface), and in the core of the ODZ. Ambient O_2_ concentrations at both the top and the core of the ODZ were below detection limit of the Seabird sensor (Table S3). The kinetics of N_2_O consumption were determined by measuring rates with varying added (^15^N)_2_O concentrations (50, 99, 198, 371, 865, and 1978 nM). Different O_2_ concentrations in incubations investigating the O_2_ tolerance of N_2_O consumption in Figs. 3,  4 were achieved prior to initiation of the experiment by varying flow rates of O_2_ and helium gases using a custom-assembled gas flow manifold with two gas flow meters on board. The O_2_ concentration for each set of exetainers was monitored by direct measurement using optical oxygen sensors with a detection limit of 0.06 µM (PyroScience GmbH, Aachen, Germany), and is shown on the *x*-axis in Fig. 3.

### Kinetics models and in situ rate estimation

Half-saturation constant (*K*_*m*_) and the maximum rate (*V*_*m*_) were determined by fitting N_2_O consumption rate and N_2_O concentration data to the Michaelis–Menten equation (Eq. (1)). *K*_*m*_ is the N_2_O concentration at which the rate (*V*) equals half of *V*_*m*_. Fitting was performed by the curve fitting tool in Matlab. The 95% confidence interval was used to determine whether a parameter is significantly different from zero. The half-inhibition constant (*K*_*i*_), analogous to *K*_*m*_, is the O_2_ concentration that causes half of the potential maximum inhibition (*I*_*m*_). *K*_*i*_ was calculated from fitting an inhibition curve (Eq. (2)). The unit of *V or V*_*m*_ is nM d^−1^, the unit of [N_2_O] or *K*_*m*_ is nM, the unit of [O_2_] or *K*_*i*_ is µM and *I*_*m*_ is unitless.

1 $$V \,=\, V_m \,\times\, \left[ {\rm{N}}_{2}{\rm{O}} \right]/\left( {\left[ {\rm{N}}_{2}{\rm{O}} \right] \,+\, K_m} \right).$$

2 $$V/V_m \,=\, 1 \,-\, I_m \,\times\, \left[ {\rm{O}}_{2} \right]/\left( {\left[ {\rm{O}}_{2} \right] \,+\, K_i} \right).$$

The in situ N_2_O consumption rate was estimated from in situ O_2_ concentration, in situ N_2_O concentration, and calculated *K*_*m*_ and *V*_*m*_. First, *K*_*m*_ in Fig. 4, measured rates (*V*) and measured [N_2_O] were used to calculate *V*_*m*_ for each depth based on Eq. (1). *K*_*m*_ and *V*_*m*_ determined in samples from the oxic layer were used to calculate in situ rates in oxic seawaters, and those determined in samples from the ODZ were used in ODZ rate estimation. Then, in situ rates were set to zero if in situ O_2_ concentrations were above a threshold level of 4.5 µM. This threshold was chosen because it was the highest oxygen concentration at which N_2_O consumption was dependent upon N_2_O concentration i.e., N_2_O consumption showed similar kinetics in response to N_2_O concentration under undetectable O_2_ concentration and at 4.5 µM O_2_ (Fig. 4e), but not at higher O_2_ concentrations. This is a conservative threshold because we did not determine the absolute highest O_2_ level that allowed N_2_O consumption.

In situ N_2_O production rate was estimated by subtracting the advection and diffusion of N_2_O from in situ consumption rates assuming a steady state (Eq. (3)). *v* (1 × 10^−7^ m s^−1^) is advection coefficient and *D* (2 × 10^−5^ m^2^ s^–1^) is the diffusivity coefficient. Coefficients and the steady-state 1-D model follow (Babbin et al.) [6].

3 $${\rm{Production}}\,{\rm{rate}} \,=	 \,\, {\rm{Consumption}}\,{\rm{rate}} \,-\, \it{v} \,\times\, \left( {\partial \left[ \rm{N_2O} \right]/\partial \rm{depth}} \right) \\ 	-\, \it{D} \,\times\, \left( {\partial ^2\left[ \rm{N_2O} \right]/\partial \rm{depth}^2} \right).$$

A mechanistic 1-D biogeochemistry model [6] was updated based on the experimental results in this study to assess the effect of different O_2_ thresholds on N_2_O predictions (Fig. S6). Briefly, the model was built upon the balance of physical and biological processes at steady state. Physical processes in the model include advection, diffusion, and gravitational sinking. Biological processes include aerobic respiration (i.e., remineralization fueled by O_2_), nitrification, and denitrification. The production of N_2_O from both nitrification and denitrification, and the consumption of N_2_O from denitrification are considered. The O_2_ threshold for N_2_O consumption is 0.3 µM in the original model and 4.5 µM in the updated model. The O_2_ threshold for N_2_O production via denitrification is 1 µM in the original model and 20 µM in the updated model. The only differences between the updated model and the original model are the O_2_ threshold values. Model code, detailed evaluation of the model and all the other parameters of the model are available in the previous study [6].

### DNA and RNA extraction, quantitative PCR (qPCR) assays, and *nosZ* microarray

These experiments were performed as previously described [15]. Briefly, DNA and RNA were extracted from Sterivex filters including four filters collected from a previous cruise in the Arabian Sea OMZ [34]. Each DNA or RNA copy number value corresponds to one Sterivex. RNA was reverse transcribed into cDNA. qPCR was used to estimate the abundance of total and transcribed *nosZ* assemblages using the nosZ1F (5′ -WCSYTGTTCMTCGACAGCCAG-3′) and nosZ1R (5′-ATGTCGATCARCTGVKCRTTYTC-3′) primer set [35]. The qPCR products were purified from agarose gels and then used as targets for microarray experiments following a previous protocol [36]. The detection limit of qPCR is 18.1 copies mL^−1^. The microarray contains 114 *nosZ* archetype probes and the sequences of all probes are published in the supplementary dataset in a previous study [15]. The fluorescence ratio of each archetype on the microarray is defined as the ratio of Cy3 to Cy5 fluorescence. Normalized fluorescence ratio (FRn) was calculated by dividing the fluorescence ratio of each archetype by the maximum fluorescence ratio on the same microarray. FRn is used as a proxy for the relative abundance of each *nosZ* archetype. FRn was used to determine the top five most abundant archetypes in each sample. Detrended correspondence analysis was performed on FRn to analyze the community composition of N_2_O-consuming assemblages using the vegan package in R (version 3.6.0). Identification of *nosZ* sequences is limited by the probe selection on the array and the larger database of *nosZ* sequences now available might help identify the oxic *nosZ* more precisely. Nonetheless, the limited database represented on the array sufficed to detect significant differences among samples and to identify phylogenetic affinities of OMZ *nosZ* genes.

## Supplementary information

Supplemental Materials
