# Supplementary material for: Microbial N2O consumption in and above marine N2O production hotspots
Source: ISME J. 2020 Dec 21;15(5):1434–44. doi: 10.1038/s41396-020-00861-2 (PMC8115077; doi:10.1038/s41396-020-00861-2)
Supplement: Supplementary file 1 — Supplemental Materials [file 41396_2020_861_MOESM1_ESM.pdf]

Supplementary Materials for

Microbial N_2O consumption in and above marine N_2O production hotspots

Xin Sun[1]*, Amal Jayakumar[1], John C. Tracey[1], Elizabeth Wallace[1], Colette L. Kelly[2], Karen L. Casciotti[2], Bess B. Ward[1]

[1]Department of Geosciences, Princeton University, Princeton, NJ, 08544, USA.

[2]Department of Earth System Science, Stanford University, Stanford, CA, 94305, USA.

*Correspondence to: xins@princeton.edu

Table S1. *In situ* oxygen concentrations and estimated N_2O consumption rates at oxic depths based on measured rates (Fig. 1c, f, i) corrected for N_2O substrate kinetics (Fig. 4b) but not corrected for *in situ* O_2 concentrations (Seabird). The rate at 90 m was used as *in situ* rate because its *in situ* O_2 (4.4 µM) was lower than the threshold 4.5 µM.

**Station**	**Depth (m)**	**Estimated Rate (nM d^{-1})**	**Seabird O_2 (µM)**
PS1	30	0.7	199.0
PS1	60	1.8	108.6
PS1	70	10.0	21.7
PS2	60	2.8	173.9
PS2	75	4.3	46.4
**PS2**	**90**	**2.7**	**4.4**
PS3	16	4.8	89.9
PS3	25	10.3	27.5

Table S2. Measured N_2O consumption rates, standard errors (SE) and r^2 values of the linear regression. 'NA' indicates that no linear increase could be found in the time course.

Station	Depth (m)	Rate (±SE) (nM d^{-1})	r^2
PS1	30	3.1±1.5	0.576
PS1	60	1.2±0.4	0.753
PS1	70	5.1±1.3	0.686
PS1	90	0	NA
PS1	100	1.3±0.6	0.726
PS1	110	0	NA
PS1	150	0	NA
PS1	260	1.7±0.5	0.596
PS1	500	0	NA
PS1	1000	0	NA
PS2	60	2.6±1.0	0.627
PS2	75	2.2±1.3	0.415
PS2	90	1.1±0.9	0.310
PS2	120	1.2±0.8	0.259
PS2	150	0.9±0.6	0.260
PS2	200	0	NA
PS2	250	2.9±1.4	0.532
PS2	300	0	NA
PS2	500	1.2±0.2	0.867
PS2	850	4.0±2.7	0.355
PS3	16	2.2±0.7	0.698
PS3	25	2.5±1.0	0.626
PS3	35	0	NA
PS3	45	0.9±0.3	0.441
PS3	60	2.6±2.0	0.200
PS3	70	0	NA
PS3	100	0	NA
PS3	160	0	NA
PS3	250	0.7±0.6	0.146
PS3	800	0	NA

Table S3. *In situ* oxygen concentrations at depths where samples were taken for O_2 tolerance (Fig. 3) and N_2O kinetics (Fig. 4) experiments in the ETNP OMZ.

Station	Feature	Depth (m)	Seabird O_2 (µM)	O_2 (µM) in Fig. 4*
PS1	Oxic layer	40	198.8	260.0
PS2	Oxic layer	60	176.8	291.9
PS2	Oxic-anoxic interface	106	below detection	12.9
PS2	Anoxic ODZ core	250	below detection	8.4
PS3	Oxic layer	16	100.1	342.0
PS3	Oxic-anoxic interface	39	below detection	4.5
PS3	Anoxic ODZ core	160	below detection	8.1

* final O_2 concentrations in incubations with O_2 addition for N_2O kinetics in Fig. 4.

Table S4. Top five total (DNA) *nosZ* archetypes at each depth in the ETNP OMZ.

	Archetypes	Origin of each archetype	FRn %
**PS1-30m**	NosZ46	uncultured clone from marsh	15.7%
	WNZ16	uncultured clone from soil	14.1%
	WNZ21	*Anaeromyxobacter dehalogenans* from soil	11.7%
	WNZ42	uncultured clone from soil	10.8%
	NosZ15	*Achromobacter xylosoxidans A8* from soil	7.2%
		**The sum of the top five archetypes**	**59.35%**
**PS1-260m**	WNZ21	*Anaeromyxobacter dehalogenans from soil*	11.5%
	WNZ16	uncultured clone from soil	11.4%
	WNZ42	uncultured clone from soil	8.7%
	WNZ23	uncultured clone from soil	6.0%
	WNZ34	uncultured clone from soil	5.9%
		**The sum of the top five archetypes**	**43.55%**
**PS2-60m**	WNZ29	*Anaeromyxobacter dehalogenans* from soil	6.8%
	WNZ36	Complete genome from marine hot spring	6.4%
	WNZ16	uncultured clone from soil	5.9%
	WNZ21	*Anaeromyxobacter dehalogenans* from soil	5.1%
	WNZ24	uncultured clone from soil	4.7%
		**The sum of the top five archetypes**	**28.80%**
**PS2-250m**	WNZ21	*Anaeromyxobacter dehalogenans* from soil	5.5%
	WNZ29	*Anaeromyxobacter dehalogenans* from soil	5.3%
	NosZ56	*Oligotropha carboxidovorans* from wastewater	4.9%
	WNZ16	uncultured clone from soil	4.7%
	NosZ21	uncultured clone from marsh	4.5%
		**The sum of the top five archetypes**	**25.06%**
**PS3-16m**	WNZ21	*Anaeromyxobacter dehalogenans* from soil	7.3%
	WNZ29	*Anaeromyxobacter dehalogenans* from soil	6.0%
	WNZ16	uncultured clone from soil	5.6%
	WNZ36	Complete genome from marine hot spring	4.9%
	NosZ21	*uncultured clone from marsh*	4.2%
		**The sum of the top five archetypes**	**27.92%**
**PS3-250m**	WNZ29	*Anaeromyxobacter dehalogenans* from soil	7.0%
	NosZ21	uncultured clone from marsh	5.4%
	WNZ36	Complete genome from marine hot spring	4.8%
	WNZ16	uncultured clone from soil	4.1%
	WNZ31	*Desulfomonile tiedjei* from sewage sludge	3.8%
		**The sum of the top five archetypes**	**25.15%**

Table S5. Top five active (RNA) *nosZ* archetypes at each depth in the ETNP OMZ.

	Archetypes	Origin of each archetype	FRn %
**PS1-30m**	WNZ16	uncultured clone from soil	7.3%
	WNZ1	uncultured clone from soil	6.9%
	WNZ21	*Anaeromyxobacter dehalogenans* from soil	5.8%
	NosZ15	*Achromobacter xylosoxidans A8* from soil	4.4%
	NosZ24	*Rhodopseudomonas palustris*	4.3%
		**The sum of the top five archetypes**	**28.69%**
**PS1-260m**	NosZ69	*Pseudomonas aeruginosa*	9.6%
	WNZ11	uncultured clone from contaminated groundwater	9.0%
	WNZ16	uncultured clone from soil	8.7%
	NosZ24	*Rhodopseudomonas palustris*	8.5%
	WNZ21	*Anaeromyxobacter dehalogenans* from soil	7.9%
		**The sum of the top five archetypes**	**43.72%**
**PS2-60m**	WNZ16	uncultured clone from soil	5.9%
	WNZ5	uncultured clone from soil	5.8%
	WNZ17	uncultured clone from soil	5.5%
	NosZ21	uncultured clone from marsh	5.2%
	WNZ21	*Anaeromyxobacter dehalogenans* from soil	5.1%
		**The sum of the top five archetypes**	**27.57%**
**PS2-250m**	WNZ1	uncultured clone from soil	10.1%
	WNZ42	uncultured clone from soil	9.6%
	NosZ29	uncultured clone from marsh	6.8%
	WNZ21	*Anaeromyxobacter dehalogenans* from soil	5.7%
	WNZ11	uncultured clone from contaminated groundwater	5.7%
		**The sum of the top five archetypes**	**37.91%**
**PS3-16m**	WNZ21	*Anaeromyxobacter dehalogenans* from soil	8.2%
	NosZ6	uncultured clone from marsh	6.1%
	WNZ16	uncultured clone from soil	5.7%
	NosZ24	*Rhodopseudomonas palustris*	4.4%
	WNZ42	uncultured clone from soil	4.3%
		**The sum of the top five archetypes**	**28.73%**
**PS3-250m**	WNZ16	uncultured clone from soil	10.4%
	WNZ21	*Anaeromyxobacter dehalogenans* from soil	9.4%
	NosZ24	*Rhodopseudomonas palustris*	8.0%
	NosZ45	uncultured clone from marsh	7.1%
	NosZ15	*Achromobacter xylosoxidans A8* from soil	5.3%
		**The sum of the top five archetypes**	**40.31%**

Table S6. Top five total (DNA) *nosZ* archetypes at each depth in the Arabian Sea OMZ.

	Archetypes	Origin of each archetype	FRn %
**10m**	WNZ29	*Anaeromyxobacter dehalogenans* from soil	5.8%
	WNZ31	*Desulfomonile tiedjei* from sewage sludge	3.8%
	WNZ21	*Anaeromyxobacter dehalogenans* from soil	3.7%
	WNZ36	Complete genome from marine hot spring	3.6%
	NosZ48	uncultured clone from marsh	3.4%
		**The sum of the top five archetypes**	**20.44%**
**60m**	WNZ29	*Anaeromyxobacter dehalogenans* from soil	7.4%
	WNZ21	*Anaeromyxobacter dehalogenans* from soil	5.8%
	WNZ16	uncultured clone from soil	5.7%
	WNZ36	Complete genome from marine hot spring	4.7%
	WNZ34	uncultured clone from soil	4.0%
		**The sum of the top five archetypes**	**27.53%**
**102m**	NosZ15	*Achromobacter xylosoxidans* A8 from soil	10.7%
	NosZ24	*Rhodopseudomonas palustris*	8.4%
	WNZ21	*Anaeromyxobacter dehalogenans* from soil	6.1%
	WNZ16	uncultured clone from soil	4.7%
	NosZ20	*Pseudomonas brassicacearum*	4.2%
		**The sum of the top five archetypes**	**34.11%**
**150m**	NosZ71	*Shewanella denitrificans*	8.3%
	WNZ16	uncultured clone from soil	7.0%
	WNZ21	*Anaeromyxobacter dehalogenans* from soil	6.8%
	NosZ8	uncultured clone from marsh	6.7%
	WNZ11	uncultured clone from contaminated groundwater	3.9%
		**The sum of the top five archetypes**	**32.73%**

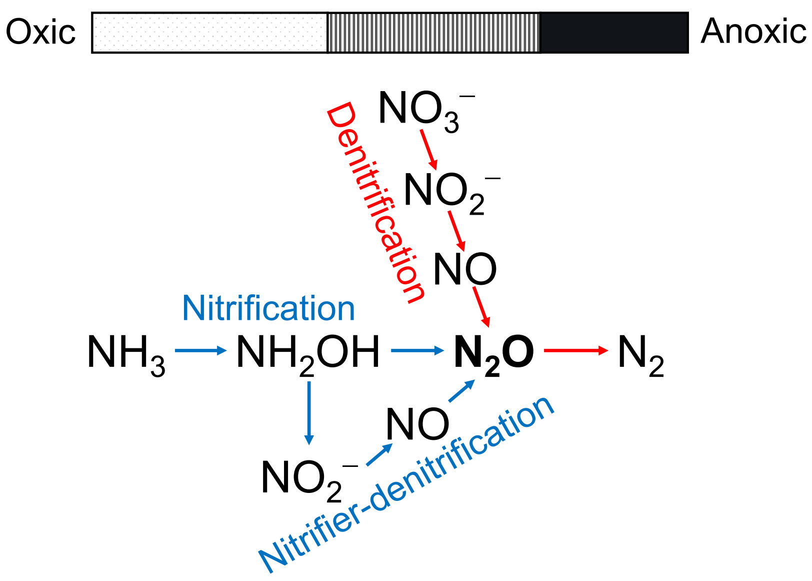

Figure S1. Pathways of microbial N_2O production and consumption. This figure is modified from [1].

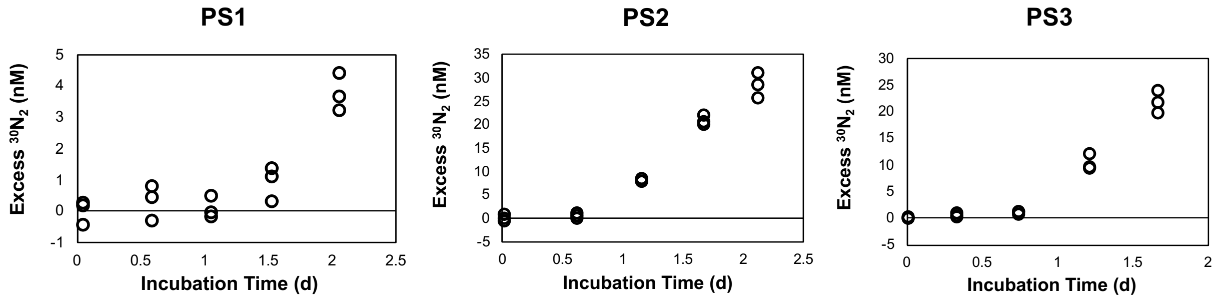

Figure S2. Time-course incubations from the oxic layer at stations PS1, PS2 and PS3. These samples were purged with helium and the total N_2O concentration was 99 nM.

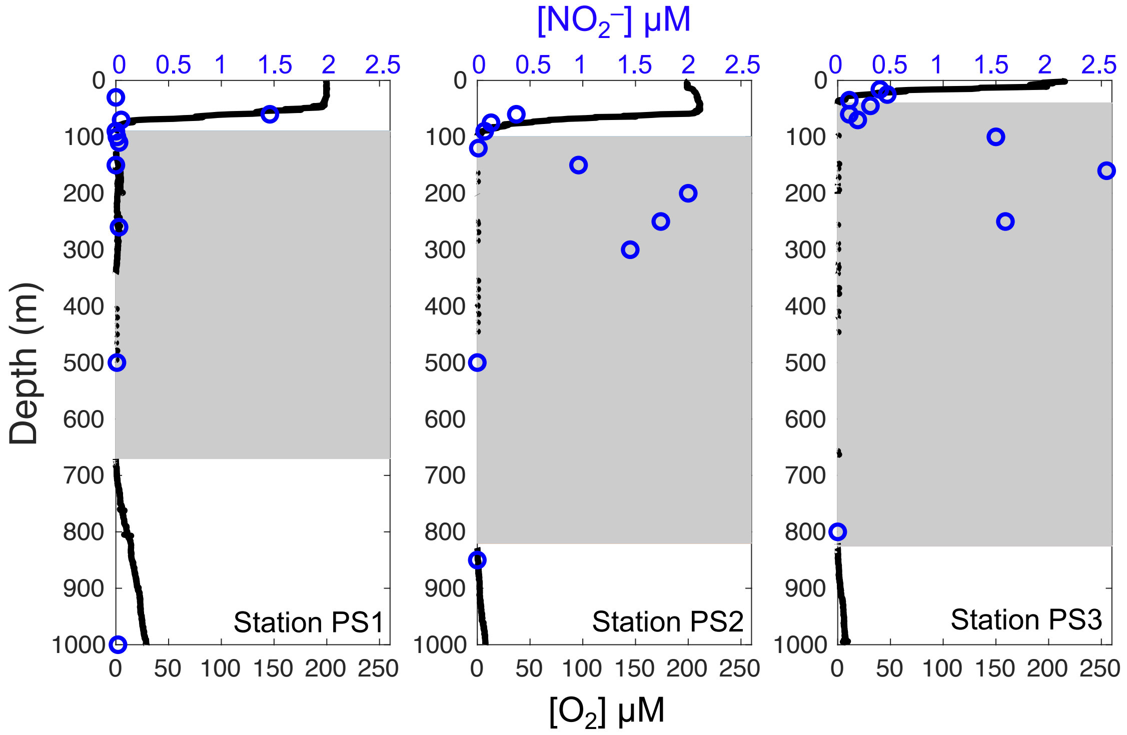

Figure S3. Depth profiles of nitrite (blue circles) and oxygen (black lines) concentrations at the three stations. Shaded areas indicate the location of the anoxic ODZ**.**

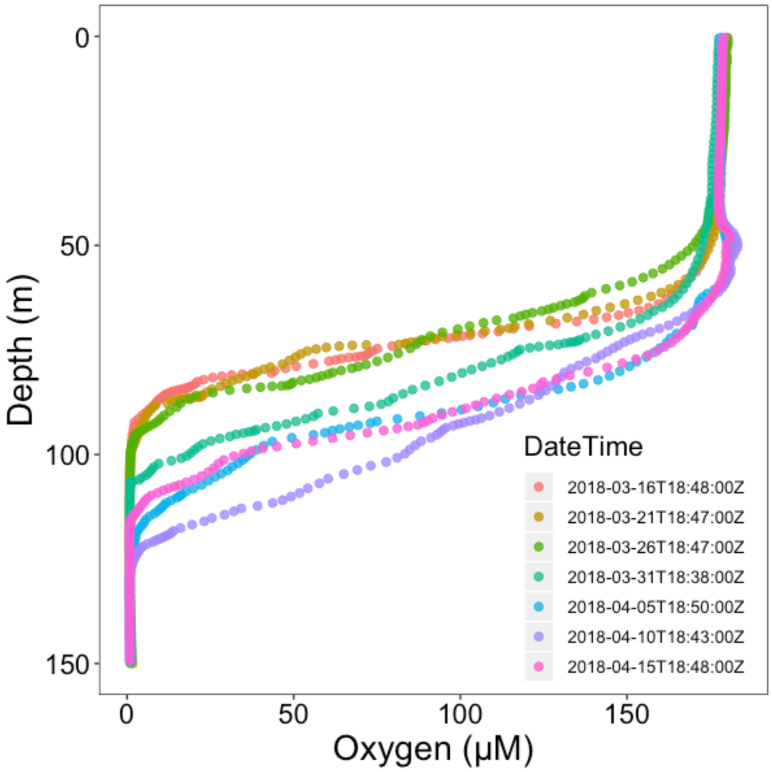

Figure S4. O_2 profiles based on one Argo float at a station (13.1 N, 108.4 W) between our sampling stations PS2 and PS3 in the ETNP OMZ. These data were collected in the same month that our samples were collected. Color indicates the date and time of data collection. The 20 m interval (100–120 m) varied from nearly 100 μM to below detection within two weeks.

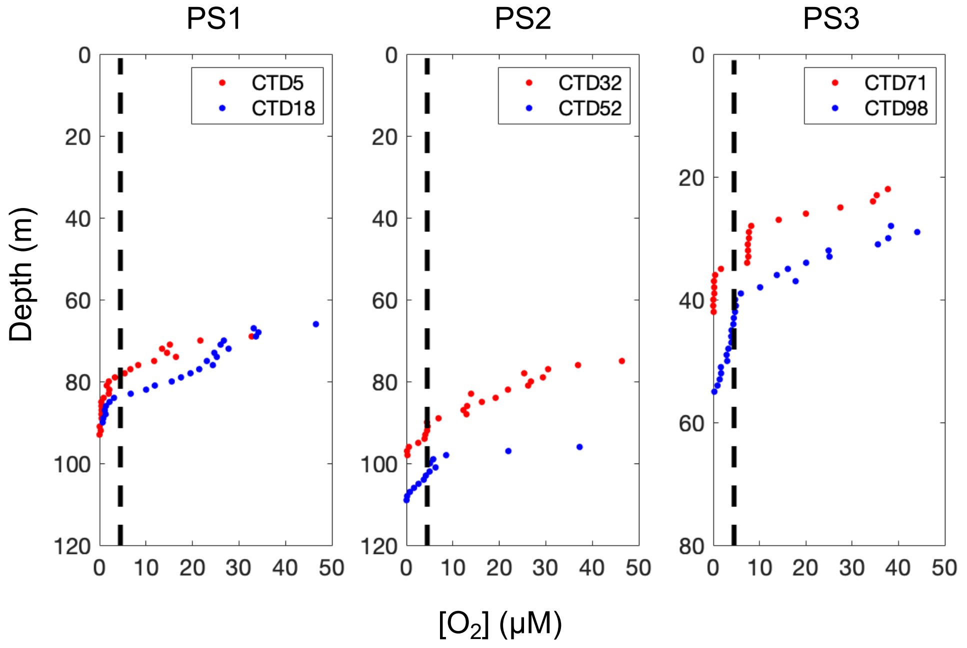

Figure S5. O_2 Profiles at stations PS1, PS2 and PS3 based on Seabird CTD. Red and blue indicate two different casts conducted within one week at the same station. Dashed black lines indicate 4.5 μM O_2.

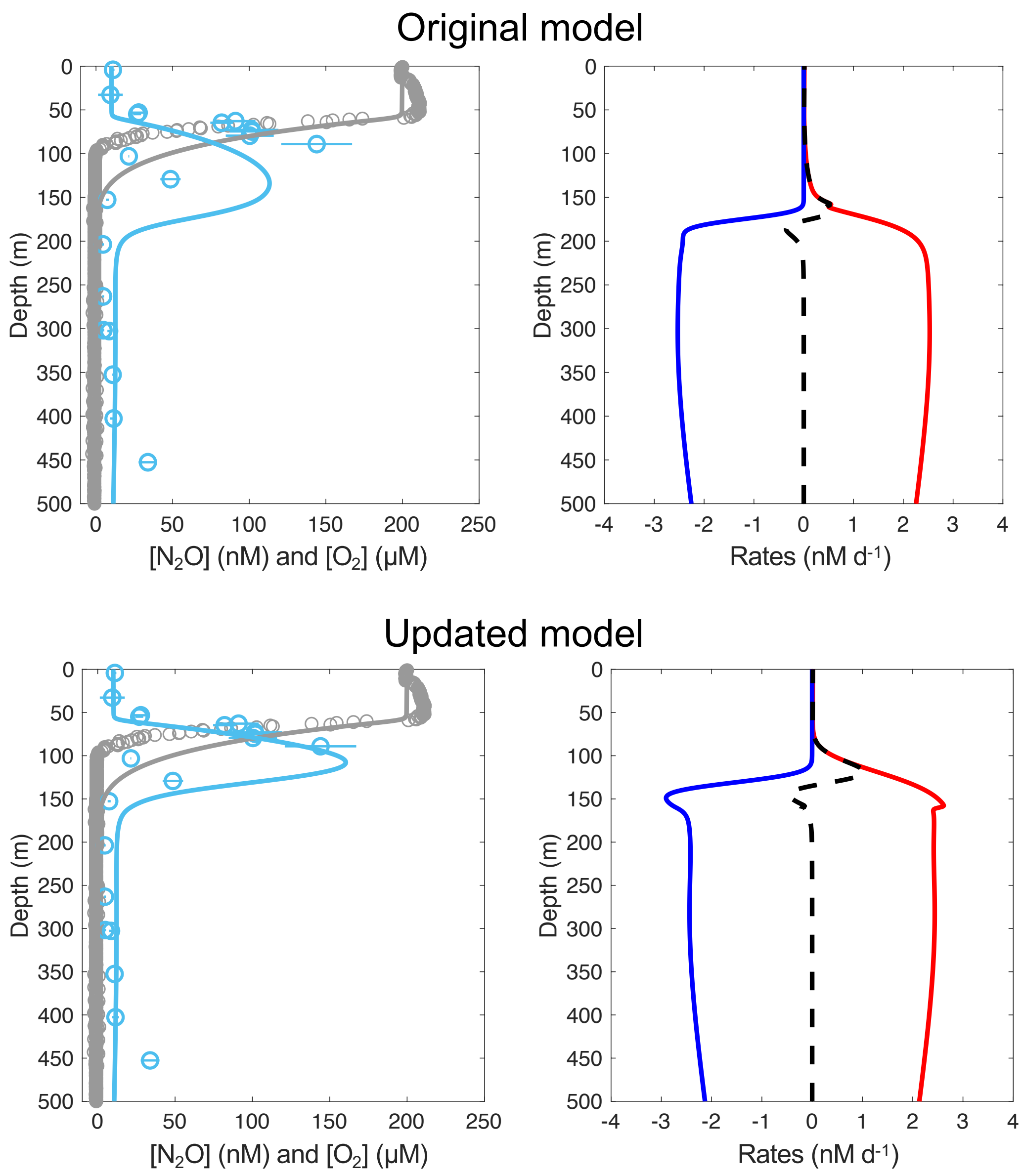

Figure S6. Comparison of results from a mechanistic 1-D biogeochemistry model [2] **with different O_2 thresholds for N_2O cycling.** Model code and detailed evaluation of the model are available in the previous study [2]. The only changes here are the O_2 thresholds. The O_2 threshold for N_2O consumption is 0.3 µM in the original model and 4.5 µM in the updated model. The O_2 threshold for N_2O production via denitrification is 1 µM in the original model and 20 µM in the updated model. The updated parameter is consistent with high tolerance of N_2O production via denitrification ($NO_3^- \rightarrow N_2O$) to O_2 [3, 4]. Left panel: modeled N_2O (light blue lines) and O_2 (grey lines) concentrations, measured N_2O (light blue open circles) and O_2 (grey open circles) concentrations from the open ocean station PS2. Right panel: N_2O production (blue), consumption (red) and net rates (black).

References

1. Bourbonnais A, Letscher RT, Bange HW, Échevin V, Larkum J, Mohn J, et al. N2O production and consumption from stable isotopic and concentration data in the Peruvian coastal upwelling system. *Global Biogeochem Cycles* 2017; **31**: 678–698.
2. Babbin AR, Bianchi D, Jayakumar A, Ward BB. Rapid nitrous oxide cycling in the suboxic ocean. *Science* 2015; **348**: 1127–1129.
3. Frey C, Bange HW, Achterberg EP, Jayakumar A, Löscher CR, Arévalo-Martínez DL, et al. Regulation of nitrous oxide production in low-oxygen waters off the coast of Peru. *Biogeosciences* 2020; **17**: 2263–2287.
4. Ji Q, Buitenhuis E, Suntharalingam P, Sarmiento JL, Ward BB. Global Nitrous Oxide Production Determined by Oxygen Sensitivity of Nitrification and Denitrification. *Global Biogeochem Cycles* 2018; **32**: 1790–1802.
